# Cancer Stem Cells and Neuroblastoma: Characteristics and Therapeutic Targeting Options

**DOI:** 10.3389/fendo.2019.00782

**Published:** 2019-11-19

**Authors:** Veronica Veschi, Francesco Verona, Carol J. Thiele

**Affiliations:** ^1^Department of Surgical, Oncological and Stomatological Sciences, University of Palermo, Palermo, Italy; ^2^Cell and Molecular Biology Section, Pediatric Oncology Branch, Center for Cancer Research, National Cancer Institute, Bethesda, MD, United States

**Keywords:** cancer stem cells, neuroblastoma, anti-cancer therapies, STAT3, mesenchymal stem cells

## Abstract

The majority of embryonal tumors or childhood blastomas derive from pluripotent progenitors or fetal stem cells that acquire cancer stem cell (CSC) properties: multipotency, self-renewal ability, metastatic potential, chemoresistance, more pronounced levels of drug transporters, enhanced DNA-damage repair mechanisms, and a quiescent state. Neuroblastoma (NB) is considered a neuroendocrine tumor and is the most common extracranial neoplasm in children. NB pathogenesis has frequently been associated with epigenetic dysregulation and a failure to implement a differentiation program. The origin, characteristics, and isolation of the CSC subpopulation in NB are still incompletely understood, despite the evidence that this cell subset contributes to disease recurrence and acquired resistance to standard therapies. Here, we summarize the literature regarding the isolation and characterization of CSCs in NB over the past decades, from the early recognition of the expression of stem cell factor (SCF) or its receptor c-KIT to more recent studies identifying the ability of G-CSF and STAT3 to support stem cell-like properties in NB cells. Additionally, we review the morphological variants of NB tumors whose recent epigenetic analyses have shed light on the tumor heterogeneity so common in NB. NB-derived mesenchymal stem cells have recently been isolated from primary tumors of NB patients and associated with a pro-tumorigenic role in the tumor microenvironment, enabling immune escape by tumors, and contributing to their invasive and metastatic capabilities. In particular, we will focus on epigenetic reprogramming in the CSC subpopulation in NB and strategies to target CSCs in NB.

## Embryonal Tumors And Pluripotent Progenitors

Embryonal tumors (ETs) can be divided into ETs of the central nervous system (CNS) in infants, such as medulloblastoma, medulloepitheliomas, atypical teratoid/rhabdoid tumors, primitive neuroectodermal tumors (PNETs) that occur in brain and spinal cord, neuroblastoma of CNS, embryonal tumors with multilayered rosettes, and other ETs or childhood blastomas that are derived from embryonal or fetal stem cells, such as neuroblastoma, retinoblastoma, rhabdomyosarcoma, nephroblastoma-Wilms' Tumor, ependymoma, and hepatoblastoma (1).

Their origin from pluripotent progenitors confers a high level of heterogeneity to these tumors, which is often the major hurdle for effective treatment. Recently, it has been demonstrated that by using a human pluripotent stem cell (hPSC)-derived tumor model in various ETs, particularly atypical teratoid/rhabdoid tumors (AT/RT), the presence of an embryonic stem cell (ESC)-like signature is associated with histology and poor prognosis of these tumors (2). These findings suggest that the activation of an early embryonic program in the hPSC progenitors may govern the genetic and epigenetic changes leading to the alteration of the differentiation potential of ETs.

In the next paragraphs, we will review one particular ET, neuroblastoma (NB) tumor, and its origin from pluripotent stem cells. We will first define cancer stem cells (CSCs) and their principal characteristics that contribute to chemoresistance in NB, such as plasticity and dormant phenotype, and we will then focus on the origin and isolation of CSCs in NB.

### CSCs and Chemoresistance

CSCs are a subpopulation of neoplastic cells characterized by asymmetrical division: a CSC divides into two daughter cells, of which one will remain a CSC (self-renewal) while the other is competent to develop into a differentiated neoplastic cell. These heterogeneous differentiated cells form the bulk of the tumor (3). Chemotherapy is an effective treatment modality to reduce or control tumor growth for many cancer patients. Many of the early chemotherapeutic drugs were cytotoxic molecules whose mechanism of action included blockade of DNA synthesis, interference with nuclear or cytosolic microtubule dynamics, or inhibition of enzymes involved in metabolism (4, 5). However, studies have shown that cancer cells, while initially sensitive, may develop resistance due to the overexpression of multidrug resistance proteins (MDR), drug efflux pumps, the accumulation of mutations in drug target genes (topoisomerase, TP53), and/or the activation of anti-apoptotic pathways (6, 7). Resistance to cytotoxic drugs may also be attributable to a subpopulation of tumor cells that is inherently not sensitive to the cytotoxic drug and the fact that many cancer stem cells have a low proliferative rate (8, 9). A recent study on a genetically engineered mouse model of glioblastoma showed that quiescent CSCs survived treatment with temozolomide and differentiated into highly proliferative cells capable of regenerating the tumor (10). Thus, CSCs may develop resistance to chemotherapy, and thus strategies using only a single cytotoxic treatment may fail, reinforcing the concept that multimodal or targeted approaches are necessary. A recent review (11) gives a more detailed description of the characteristics of CSCs and the genetic, epigenetic, and metabolic mechanisms that contribute to their chemoresistance.

### Plasticity and Dormant Cells: CSC Characteristics of Chemoresistance

Plasticity is the capacity of cells to acquire different phenotypes. This characteristic has been shown during the terminal differentiation of adult tissues, which undergo physiologic or pathologic chronic stress conditions, and maintain the ability to change their phenotype. Plasticity is important for tissue regeneration and response to environmental stimuli, but it can be exploited by cancer cells. Cellular phenotypic changes are not only intrinsic to the cancer cell during tumor progression but can be influenced by the tumor microenvironment (signaling molecules, stromal cells, vascular supply, and therapeutic agents). The phenome of a cell is the sum of all its phenotypic traits and may arise due to alterations in the epigenetic thresholds caused by genetic alterations driving tumor progression (12). They may be associated with the induction of a more aggressive cancer cell or a subpopulation resistant to chemotherapeutic drugs, able to escape the immune system and with metastatic potential.

The contribution of plasticity to tumor initiation has been described in terms of two principal models. The first suggests that different tumor types arise from different cancer cells. For instance, cancers that occur in liver can be distinguished in hepatocellular carcinoma (HCC) and cholangiocarcinoma (CC) and may derive from the hepatocytes or from the cholangiocytes, respectively. The second hypothesizes that the same original cancer cell may promote different malignant cancer phenotypes through a plasticity process due to genetic alterations and dynamic epigenetic changes (13). Intrinsic cellular plasticity has also been considered as a contributor to cancer dormancy, a mechanism fundamental to the metastatic process.

The time period during which patients do not show any symptoms before cancer relapse is known as cancer dormancy. Field experts have elaborated two general models of dormancy. The first, tumor mass dormancy, is characterized by an interruption of overall tumor growth due to the balance between proliferation and cell death, which may be associated with angiogenic and immunologic dormancy (14–17). The second category, known as cellular dormancy or solitary cell dormancy, refers to the capacity of a single cancer cell to temporarily arrest its cell cycle (18–20). Plasticity and dormancy are two fundamentally interconnected biological states in cancer stem cells, and they affect the CSC chemo-resistance and metastatic potential through metabolic reprogramming, epigenetic alterations, and complex interactions with the immune system.

Of note, in metastatic NB tumors, aggressive cellular clones with CSC characteristics derived from different metastatic sites retain plasticity and adaptive stemness after sequential changes in culture conditions *ex vivo*. These clones undergo dynamic and reversible stem cell-related molecular transitions in response to tumor microenvironment stimuli, as demonstrated by a comprehensive study of epithelial-mesenchymal transition (EMT)- and stemness-associated molecules based on functional and transcriptomic data (21). The continuous molecular rearrangements in these metastatic clones may have determined the heterogeneity of high-risk NB tumors and the occurrence of poor clinical behavior. Interestingly, it has been demonstrated that *in vitro* switching between two cellular phenotypes maintaining stem-like properties could be responsible for chemoresistance and functional heterogeneity of NB. These two cellular states of the murine, Neuro2a, and human, IMR-32 and SK-N-SH, NB cell lines show different capabilities in terms of anchorage-dependent or independent growth and distinct molecular signatures upon different culture conditions *in vitro*. This can be defined as a new form of “reversible adaptive plasticity,” supporting the dynamic nature of NB CSCs and their flexibility to interconversion between different cellular states (22). These recent findings are not surprising considering that the plasticity of NB cells was discovered a long time ago, but previous findings were mostly related to their ability to differentiate along the fetal ganglionic lineage between the neural crest stem cell progenitors and the adrenal medullary precursor cells (23).

Another mechanism of chemoresistance in NB cells is the acquisition of a dormant phenotype that may last decades and may account for late relapse, as was reported in a clinical case report of a patient affected by metastatic NB after 52 years of apparent dormancy (24, 25). MYCN oncogene contributes to the maintenance of stemness in NB CSCs, contributing to their plasticity and chemoresistance. In fact, because of their dynamic response to the tumor microenvironment, CSCs can escape from anti-tumor therapies by entering a dormant state, defined as the G0/G1 quiescent phase of the cell cycle (26).

## Origin And Isolation OF Cancer Stem Cells In Neuroblastoma

**Figure 1:**
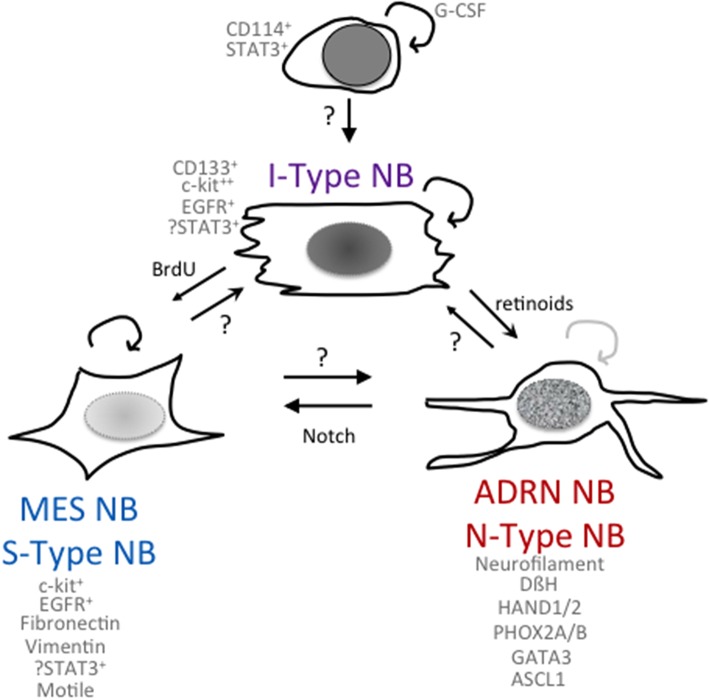
Phenomes of NB cells with cancer stem cell or tumor-initiating properties. Schematic of potential relationships among NB cells with differing phenotypes and functionalities.

Neuroblastoma (NB), one of the most common childhood extracranial solid tumors, derives from embryonic neural crest cells. NB frequently occurs along the chain of sympathetic neural tissue along the spinal cord and also in the adrenals, since the adrenal medullary cells are neural crest-derived. The most aggressive tumors display amplification of the MYCN oncogene and TERT rearrangements, which is associated with poor survival, even in localized disease. NB has also been considered a neuroendocrine tumor as it derives from neural crest cells (nervous system) and is often localized in hormone-producing organs such as the adrenal gland (endocrine system). This particular origin or localization and its heterogeneity and failure to differentiate, represent the main characteristics of NB and are thought to contribute to the lack of efficacy of multimodal therapeutic approaches. Thus, identifying the properties of the most aggressive NB cancer stem cell (CSC) or tumor-initiating cells (TICs) may pave the way to promising therapeutic strategies. In the next paragraph, we will summarize the history of the isolation of NB CSCs (Figure 1).

### What Is Already Known About the Origin and Isolation of CSCs in Neuroblastoma?

Some 30 years ago, studies indicated the presence of a subpopulation of stem cell-like progenitors or tumor-initiating cells in NB tumors. Stem cell factor (SCF) and its receptor c-kit are simultaneously expressed in several NB cell lines and tumors (27). SCF and c-kit is a key signaling pathway that is involved in many fundamental biological processes, such as gametogenesis, hematopoiesis, and melanogenesis, and has also been shown to promote cancer growth in tumors such as acute myeloid leukemia, small cell lung carcinoma, and breast carcinoma (28, 29). By treating NB cell lines (SK-N-BE and SH SY5Y) with an anti-c-kit antibody, tumor growth but not differentiation was reduced, thus implicating an autocrine regulatory loop of SCF/c-kit in the growth of NB cells (27). The trophic role of SCF ligand on neural crest cells has been demonstrated by studies on human and animal models with an induction of the outgrowth of the c-kit positive neurites. However, the role of c-kit in NB differentiation is unclear despite findings that c-kit mRNA expression levels were variably expressed in different NB cell subsets. Although NB cells are sensitive to imatinib mesylate (gleevac), a small molecule inhibitor of protein tyrosine kinases including c-kit, it was unclear whether this was a generalized decrease in NB cell growth or resulted from the inhibition of a NB stem-like cell (30).

The Biedler lab first noted heterogeneity in NB cell lines with the isolation of morphologic variants from NB cell lines (31) that had distinct gene expression profiles and were capable of interconversion. N-type (neuroblastic) sympathoadrenal neuroblasts have short neuritic processes, S-type (substrate-adherent) cells had glial or Schwannian-like characteristics, and I-type (intermediate) cells express features of both N- and S-type cells (Figure 1). They found the I-type cells represent the most aggressive subpopulation within a tumor and could give rise to N- and S-type NB cells (31, 32). I-type NB cells show properties of stem-like cells, including the expression of stem cell markers such as CD133 and c-kit, and advanced stage NB tumors had higher levels of expression of cells with I-type characteristics than tumors from lower stages. Transcriptional profiling (33) of the I-type cells compared with N- or S-type cells revealed elevated levels of stemness markers including: *Notch*, whose overexpression impairs the neural/glial differentiation maintaining the stem cell and the malignant potential of these cells; PIGF2, the placental growth factor that induces angiogenesis; GPRC5C, which is highly expressed in embryonic stem cells and TRKB and LNGFR, which have been shown to increase the survival, invasiveness, and chemoresistance of this subpopulation (34, 35). Thus, the I-type NB cells have stem cell-like features, with both the potential for self-renewal and for multi-lineage differentiation or bi-directional differentiation into N- or S-type NB cells. This supports the hypothesis that a stem cell or tumor-initiating cell contributes to the growth of NB tumors (32). All of these three components have been found in primary NB tumors and/or isolated from bone marrow aspirates, suggesting that the proportions of these different cell types may play a role in affecting the clinical behavior of the NB tumor. In a small selected cohort of primary NB tumors, a higher fraction of cells with I-type markers was found in tumors from patients that eventually relapsed as compared to tumors that were progression-free (32).

The tumor microenvironment can influence tumor stemness, and in particular, it has been demonstrated that hypoxia enriches stem-like populations and increase their invasive capacity in NB. Specifically, upon the exposure to an “injured conditioned medium” derived from bone marrow stromal cells exposed to hypoxia and oxidative stress, a highly tumorigenic fraction of cells (functionally defined by side population = SP) were found to express high levels of the stemness marker Oct4 and migrate to this conditioned medium *in vitro* and to hypoxic zones in *in vivo* xenograft models. The SP represent a subset of cells isolated from several different tumors endowed with CSC-like properties. The ability of this SP fraction to migrate to the hypoxic/ischemic region of NB tumor suggests that the hypoxic tumor microenvironment may represent the ideal niche for these cells and also for the cancer stem cell (CSC) fraction dynamically subjected to alternative phases of acute and chronic hypoxia, which mimic stress, or injury conditions (36).

These early studies on stem cell properties in NB were limited by their reliance on NB cells that had been adapted to cell culture for many years, and it was unclear how relevant they were compared to a patient's primary, chemo-refractory, or relapsed tumors. David Kaplan's research team and others isolated NB cells from primary tumors and bone marrow metastases and first maintained them in defined media. They used both molecular markers and functional assays to show that advance stage NB tumors contain a high frequency of tumor-initiating cells (TICs), cells with cancer stem cell functionalities. They noted differences between TICs isolated from NB tumors from patients with high- and low-risk clinical parameters and identified CD24 and CD34 as potential markers expressed by TICs that enabled xenograft tumor formation at a lower precursor frequency. In particular, sphere-forming cells derived from high-risk NBs exhibited a higher frequency of self-renewal and capacity to form metastatic tumors in murine xenograft models, even when 10 cells were implanted at an orthotopic location (37). To understand whether there were differences in chemosensitivity, they performed a high-throughput small-molecule screen using these TICs. Two compounds were shown to selectively inhibit NB TICs (DECA-14 and rapamycin) at nanomolar concentrations *in vitro* and to dramatically reduce tumor growth *in vivo*. DECA-14 is a dequalinium analog, C-14 linker, which is an antimicrobial agent frequently used in over-the-counter medicinal products such as throat lozenges or mouth wash. Interestingly, these compounds were ineffective on normal pediatric stem cells (skin-derived progenitors or SKPs) that share similar neural crest stem cell markers and have stem cell-like properties like NB TICs but are not tumorigenic. These results indicated that specific drugs could be developed that selectively inhibit a CSC population while potentially sparing a normal stem cell compartment. This is an important consideration when one considers the targeted treatment of stem cells in pediatric cancers, where it is essential to minimize effects on normal development (38). More recently, the role of c-KIT in NB tumor cells has been revisited with the finding that high levels of c-kit in NB tumors are associated with poor patient prognosis. Using primary NB tumor TICs or NB cell lines in culture, a transient population (about 5%) of NB cells express high levels of c-KIT, and these cells were found to have a higher growth rate than c-kit-low cells. *In vitro*, selected high c-kit-expressing cells re-establish in NB cell lines or NB-TIC in culture. The finding that vascular endothelial growth factor/prokineticin-1 signaling, a niche factor important in the development of enteric neural crest cells, is important for maintaining c-kit ^+^ NB cells suggests that the microenvironment of a tumor cell affects its aggressiveness. A downstream target of activated c-kit is mTOR, which may provide a mechanistic link to the selective activity of rapamycin in inhibiting NB-TICs (39).

Neural crest development is a finely regulated process that orchestrates the equilibrium between pro-stemness and pro-differentiation signals. The STAT3 signaling pathway is required for correct neural crest development, and its activation, mediated by the granulocyte colony-stimulating factor (G-CSF), has been implicated in the tumorigenesis of neural crest-derived tumors (40). Based on findings that G-CSF also played a role in the survival and differentiation of progenitor cells in the post-ischemic brain (41), the Shohet lab identified an enriched population of G-CSF receptor (G-CSFR/CD114) positive cells in chemotherapy-resistant or relapsed NB (42). These CD114^+^ cells had a transcriptome similar to early neural crest progenitors, embryonic stem cells, and induced pluripotent progenitors. Their subsequent studies identified that G-CSF via activation of STAT3 promoted NB tumorigenicity and metastasis (43, 44), raising the possibility that small molecule inhibitors of the JAK/STAT pathway that were being developed for other cancers may selectively target these highly aggressive NB tumor cells. AZD1940, a relatively selective JAK/STAT inhibitor, was shown to inhibit NB tumor cell growth *in vitro* and decrease NB xenograft growth *in vivo* (45). However, small-molecule inhibitors specific for the JAK/STAT pathway have been difficult to develop, and many have significant activities against other kinases. A specific STAT3 targeted agent is AZD9150, a 16-oligonucleotide antisense molecule targeting the 3′ region of human STAT3 and inhibiting mRNA and protein production. Systemic administration limits its effectiveness in solid tumors, but a Phase I study did show inhibition of the target STAT3 and reduced tumor growth in Diffuse Large B-Cell Lymphoma (46). In preclinical studies in NB, AZD9150 selectively inhibited cytokine-activated STAT3 signaling yet showed only a modest 20% inhibition of NB cell line growth *in vitro*. Intratumoral injections showed that AZD9150 inhibited STAT3 expression yet had little effect on the primary growth of tumor xenografts. However, utilizing a secondary tumor formation assay for stem cell activity, it was found that AZD9150-treated tumor cells had a decreased ability to initiate secondary tumor formation. Limiting dilution analyses indicated that there was a 10-fold decrease in the precursor frequency of NB tumor-initiating cells from AZD9150-treated primary tumors compared to the frequency of tumor-initiating cells in untreated primary tumors (47). Although it is not clear that the NB TIC in this study is the same as the CD114^+^ cell in previous studies, it does provide compelling evidence for the presence of a STAT3-expressing NB tumor-initiating cell.

### The Role of miRNAs and lncRNAs in the Control of NB Cell Stemness and Differentiation

miRNAs have been implicated in the regulation of stem cell maintenance and neuronal differentiation (48, 49). In several tumors, such as colorectal cancer, breast cancer, and lung cancer, it has been demonstrated that miRNAs modulate the maintenance, proliferation, and reprogramming of CSCs by targeting the principal oncogenic signaling pathways, such as the notch, Wnt/β-catenin, PI3K/AKT, and JAK/STAT pathways (50). Also, in NB tumor, it has been shown that miR-25 modulates CSC stemness through interaction with Gsk3β and activation of the Wnt pathway (51). Moreover, Hsu et al. identified a signature of 25 miRNAs differentially expressed in CD114^+^- vs. CD114^−^-expressing NB cell lines. This CD114^+^ miRNA signature is consistent with the miRNAs overexpressed in the transition of embryonic stem cell (ESC) to neuronal precursors (miR-106b, 21, 25, 30e, 598, 93), and the miRNAs repressed upon neuronal lineage differentiation (miR-25, 106b, 17, 18, 19, 20a, 143, 27) (42).

A number of miRNAs have been shown to regulate various aspects of NB cell differentiation, but a consensus as to which are critical regulators has yet to emerge. These include miRNA-449a, miR-10a, miR-10b, miR-204, miR-506-3p, and miR-124-3p, with miRNA-449a regulating the PKP4 and MFAP4 genes involved in cellular interactions and the control of neuritic extensions (52). MiR-10a is upregulated in the differentiated NB cell lineage after administration of all-trans-retinoic acid (ATRA) (53), and one mechanism may be miR-10b inhibition of Nuclear Receptor Corepressor 2 (NCOR2), a transcriptional co-repressor, which regulates the genes involved in differentiation of NB cells (54). MiR-204 decreases the expression levels of PHOXB2, which plays a central role in modulating the differentiation of NB cells (55). Zhao et al., using a functional high-content image screen, identified 14 miRNAs, including miR-506-3p and miR-124-3p, whose loss was associated with neurite outgrowth (56). Knockdown of the long non-coding RNA (lncRNA), Malat1, induces a reduction of neurite outgrowth and is associated with inhibition of the ERK\MAPK pathway, which blocks the differentiation of N2a NB cells (57).

## Mesenchymal And Adrenergic Phenotypes Of Neuroblastoma

Some 10 years ago, epigenetic alterations, mainly promoter hypermethylation and inactivation of tumor suppressor genes, were recognized to play a major role in several deadly tumors, including colon cancer and glioblastoma. Recently, a large number of mutations have been found to affect the expression/function of a large variety of proteins involved in modifying chromatin. In addition, increasing evidence shows that super-enhancer-driven core regulatory transcriptional circuits drive lineage specificity during normal development and that malignant transformation may co-opt these circuitries to drive tumor formation (58, 59). NB and other pediatric tumors have a low mutational burden, suggesting that alterations in their epigenetic regulation may be crucial mechanisms in tumor initiation and progression. This dysregulation of the epigenome may contribute to the heterogeneity and plasticity common in ETs and NB in particular. Several recent studies have dissected the NB epigenetic landscape, demonstrating a vulnerability to compounds that target epigenetic enzymes crucial for NB cell survival, proliferation, and differentiation, such as SETD8 and EZH2 (60–62).

Using patient-derived cell lines, Versteeg et al. have molecularly characterized two NB cell types, an adrenergic (ADRN) NB cell type and a mesenchymal (MES) NB cell type (Figure 1) (63), that are capable of interconversion and share properties with the N- and S-type NB cells first identified by Biedler and colleagues (see above). *In vitro* experiments on cell lines derived from the same patient showed different mRNA expression levels of the cancer stem cell marker CD133 (64, 65). CD133^−^ cells propagated as semi-attached spheres and did not migrate, while CD133^+^ cells grew attached, formed lamellipodia, and were able to migrate. Gene set enrichment analysis showed that CD133^−^ cells present an adrenergic phenotype associated with high levels of PHOX2A, PHOX2B, and DBH, typical of classic NB cells, while the CD133^+^ cells showed high levels of SNAI2, VIM (vimentin), and FN1 (fibronectin), which are typical mesenchymal cell markers (63). Using four isogenic cell lines, van Groningen et al. found 485 genes associated with an MES mRNA signature and 369 genes associated with an ADRN mRNA signature. These genes were used to evaluate 33 NB cell lines, and it was revealed that most NB cell lines clustered as ADRN or MES, although some had an intermediate phenotype between MES and ADRN. Interestingly, the signatures of the MES cells were similar to those of human neural crest-derived cell lines, suggesting that MES cells correspond to precursors of the adrenergic lineage (63). To confirm the relationship between MES and ADRN, van Groningen studied the trans-differentiation potential of the two cell types. CD133^+^ and CD133^−^ cells from the heterogeneous cell line AMC700B were sorted, and CD133^+^ was found to lack clonogenic potential but within eight passages contained almost 50% CD133^−^ cells. The trans-differentiation capacity of CD133^+^ and CD133^−^ cells *in vivo* was observed by subcutaneously injecting these cells into mice. Both CD133^+^ and CD133^−^ cells formed tumors in mice, although CD133^−^ cells were more aggressive than CD133^+^ (63). Overexpression of the homeobox transcription factor PRRX1 (63) or activation of notch in an ADRN NB cell induces transdifferentiation to an MES phenotype, which is more resistant to the cytotoxic agents used in treatment of NB (66). Specifically, van Groningen and colleagues have demonstrated that MES NB cells are more resistant *in vitro* to the commonly used drugs for the treatment of NB, doxorubicin, cisplatin, and etoposide, compared with ADRN NB cells. Furthermore, the number of PRRX1^+^ MES NB cells increases in tumors treated with standard chemotherapy and in relapsed tumors *in vivo* (63). The downstream activation of notch signaling or the overexpression of PRRX1 may be responsible for the different sensitivity to drugs of the two cellular phenotypes. However, further studies are needed to elucidate the mechanisms involved in NB cell chemoresistance, particularly in the MES phenotype.

Evidence suggests that super-enhancers play a central role in cell identity (58, 59, 67). ChIP-seq analysis for H3K27ac and H3K4me3 histone modification was performed in four MES and five ADRN NB cell lines, including three isogenic cell pairs. Epigenetic bioinformatic analyses indicated that the nine cell lines clustered in two separated groups: MES and ADRN. The same clustering was found in the three isogenic pairs. Some 286 super-enhancers were associated with the MES type, and 276 super-enhancers were linked to the ADRN-type. The genes, such as DBH, CHGA, and DLK1, associated with the ADRN-specific super-enhancers were associated with known markers of adrenergic differentiation, while genes associated with MES-specific super-enhancers included WNT5A, IGFBP2, FN1, and IL13RA1. Induced pluripotent stem cell (iPSC) research has suggested that cell identity is imposed by a core set of TFs that mutually bind one another's super-enhancers, thus creating a feed-forward loop and resulting in very high expression of their associated gene (58, 59, 67). In NB, they found 20 MES TF genes with strong MES-specific super-enhancers, for example, *MEOX1, MEOX2, SIX1, SIX4, SOX9, SMAD3*, and *WWTR1* and 18 ADRN super-enhancer-associated TF genes, including *ASCL1, EYA1, GATA3, HAND1*, and *SIX*. These super-enhancer-associated TF genes are therefore candidate master gene regulators of the two cell types. These different cell types have differing functionalities, with the ADRN cells being more sensitive to cytotoxic drugs than the MES NB cells and MES cells being more migratory than ADRN cells.

A commonality among the NB cell types with stem cell-like behavior is their enrichment in tumors from chemotherapy refractory or relapsed patients. Thus, it is imperative to study the vulnerabilities of these different cell types. Several studies have shown that N-type or ADRN NB cells are more sensitive to cytotoxic agents than MES or S-type NB cells. However, the differential expression of caspase 8 by S-type but not N-type NB cells is associated with their sensitivity to TRAIL-induced apoptosis (68). Additionally, systemic administration of IFNγ to NB patients has been shown to induce caspase 8 in their tumors and IFNγ treatment of N-type NB cells *in vitro* also induces caspase 8, rendering many NB cell lines sensitive to TRAIL-induced apoptosis (69). Given the propensity of NB cells to transdifferentiate, it will be important to understand the signaling pathways that control these conversions in order to optimize treatment modalities and relapse.

## The Role Of Mesenchymal Stem Cells

### Mesenchymal Stem Cells in NB (NB-MSCs) Isolated From NB Tumor Mass

**Figure 2:**
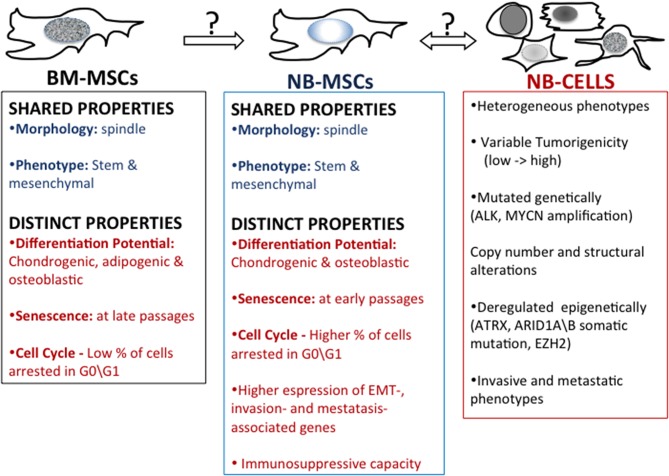
Mesenchymal stem cells in neuroblastoma. A comparison of properties of bone marrow-derived mesenchymal stem cells (BM-MSC) and mesenchymal stem cells harvested from patient's NB tumors (NB-MSC) and properties of NB tumor cells.

Recent evidence highlights the influence that cells in the tumor microenvironment play exert over tumor growth (70, 71) as well as how the tumor cells or secreted tumor factors play at distant tissues that may become sites of tumor metastases (72). Pelizzo et al. (73) proposed that mesenchymal stromal cells (MSCs) isolated from the NB tumor mass promote tumorigenesis by interacting with tumor cells and other stroma cells in the complex network of the tumor microenvironment (Figure 2). Neuroblastoma mesenchymal stem/stromal cells (NB-MSCs) were defined as cells of the tumor microenvironment isolated from seven patients with diagnoses of NB. NB-MSCs showed a spindly morphology and entered into senescence faster than BM-MSCs (control). This, together with cell cycle analyses indicating that NB-MSCs show a higher number of cells arrested in G0-G1, suggests that cells are primed for “dormancy.” Dormant tumor cells are characterized by G0/G1-phase arrest and resistance to chemotherapeutic drugs (74). Flow cytometric analyses showed that NB-MSCs have the same level of expression of stem markers Sox2, Oct3\4, and Nanog as BM-MSCs. This suggests that NB-MSCs retain pluripotent potential in the tumor microenvironment. Moreover, NB-MSCs and BM-MSCs share similar levels of expression of membrane markers typical of MSC cells, such as CD73, CD90, CD105, and HLA-I. Since MSCs have the potential to differentiate into many different cell types, Veschi et al. studied the differentiation potential of NB-MSCs (73). NB-MSCs were able to differentiate into osteocytes and chondrocytes but not into adipocytes. This data suggests that NB-MSCs are both morphologically and functionally altered.

MSCs regulate immunity by interacting with innate immune cells (including macrophages, NK cells, and dendritic cells) and adaptive immune cells (including B and T cells) (75, 76). The influence of NB-MSCs on the immune system was studied by co-culturing NB-MSCs with T lymphocytes obtained after treatment with phytohemagglutinin (PHA-P). NB-MSC exerted an anti-proliferative effect on immune cells compared with BM-MSCs. The differing developmental potential and functional effects on T-cells suggest that NB-MSCs have the potential to impact the tumor microenvironment in a manner distinct to BM-MSCs (71). Gene expression profile analysis revealed that NB-MSCs isolated from tumor samples derived from NB patients were enriched in EMT-associated genes compared to BM-MSCs. NB-MSCs showed up-regulated expression of CDH2 and MMP-9 genes. CDH2, also known as N-cadherin, favors trans-endothelial migration, and MMP-9, a crucial enzyme involved in extracellular matrix remodeling, promotes cell invasion and metastasis by collagens and fibronectin degradation. Of note, high expression levels of CDH2 and MMP-9 correlate with poor prognosis in NB tumors (77, 78). Moreover, NB-MSCs expressed higher levels of CXCR4 and reduced levels of CXCL12 compared to BM-MSCs, supporting the pivotal role of the CXCL12/CXCR4 axis in promoting NB invasiveness (73).

The tumor microenvironment and NB-MSCs play a central role in promoting the immune-escape, invasiveness, and metastatic propensity of NB cells. What role they play in the phenotypic switching of the MES and ADRN NB types remains to be delineated, as does the contribution of the tumor microenvironment to this process. Additionally, how the tumor impacts the complex networks regulating the possible switching between BM-MSCs-NB-MSCs and NB cells remains to be explored. Consideration of these events will be crucial to designing novel approaches targeted to the NB tumor cells and cells within its microenvironments that support its growth and suppress immune surveillance.

## Concluding Remarks

ETs, due to their origin from pluripotent progenitors, are resistant to standard therapies. Novel insights into the molecular mechanisms and genetic and epigenetic changes characterizing the CSC subpopulation in ETs, and in NB in particular, have been provided over the last 30 years. These findings have shed new light on potential different therapeutic applications and strategies for targeting CSCs in NB, from the use of last-generation compounds as STAT3 inhibitors to the use of stromal cells as NB-MSCs as a tool for drug delivery. Additional efforts are needed, especially for the high-risk NBs, which may represent a switch from an embryonic-like condition to malignant metastatic disease due to the co-option of early embryonic programs. Given that epigenetic programs are crucial during differentiation and development, it is easy to understand the impact of epigenetic alterations in NB, which is fundamentally a neurodevelopmental disease due to a failure of neuroblasts to differentiate. NB has been definitely considered a “stem cell tumor.” Several epigenetic modifications have been implicated in endowing stemness and self-renewal properties to NB CSCs. Moreover, recently developed epigenetic inhibitors, which may target crucial regulatory processes for the formation and maintenance of the CSC population, have entered clinical trials for pediatric tumors.

## Author Contributions

VV and CT conceptualized and wrote the manuscript. FV contributed to drafting the manuscript.

### Conflict of Interest

The authors declare that the research was conducted in the absence of any commercial or financial relationships that could be construed as a potential conflict of interest.
